# Volitional components of consciousness vary across wakefulness, dreaming and lucid dreaming

**DOI:** 10.3389/fpsyg.2013.00987

**Published:** 2014-01-02

**Authors:** Martin Dresler, Leandra Eibl, Christian F. J. Fischer, Renate Wehrle, Victor I. Spoormaker, Axel Steiger, Michael Czisch, Marcel Pawlowski

**Affiliations:** Max Planck Institute of PsychiatryMunich, Germany

**Keywords:** lucid, dreaming, sleep, volition, consciousness, metacognition

## Abstract

Consciousness is a multifaceted concept; its different aspects vary across species, vigilance states, or health conditions. While basal aspects of consciousness like perceptions and emotions are present in many states and species, higher-order aspects like reflective or volitional capabilities seem to be most pronounced in awake humans. Here we assess the experience of volition across different states of consciousness: 10 frequent lucid dreamers rated different aspects of volition according to the Volitional Components Questionnaire for phases of normal dreaming, lucid dreaming, and wakefulness. Overall, experienced volition was comparable for lucid dreaming and wakefulness, and rated significantly higher for both states compared to non-lucid dreaming. However, three subscales showed specific differences across states of consciousness: planning ability was most pronounced during wakefulness, intention enactment most pronounced during lucid dreaming, and self-determination most pronounced during both wakefulness and lucid dreaming. Our data confirm the multifaceted nature of consciousness: different higher-order aspects of consciousness are differentially expressed across different conscious states.

## INTRODUCTION

The ability to engage in volitional behavior has traditionally been closely associated with human consciousness: to freely act implies to make conscious decisions (Dijksterhuis and Aarts, 2010). However, consciousness is not an all-or-nothing phenomenon; its multiple facets differ across species, vigilance states, or health conditions. A striking variation in consciousness is experienced every day during the sleep–wake cycle: during wakefulness, human subjects are normally alert, aware of external and internal stimuli, able to reflect on their perceptions, emotions and thoughts, and capable to volitionally act according to their intentions. While most of these properties of waking consciousness fade during the process of falling asleep, many basal features of consciousness reappear during dreaming. Dream mentation may occur in all sleep stages, but is most intense and vivid during rapid eye movement (REM) sleep (Hobson et al., 2000). The dreamer perceives and interacts with a hallucinated dream environment and often experiences strong emotions (Hobson and Pace-Schott, 2002). However, typical dreaming is deficient of many higher-order aspects of consciousness: the dreaming subject experiences highly impoverished self-reflective capabilities and therefore does not recognize that he is dreaming. Instead of volitionally and systematically acting according to his intentions, the dreamer is a rather passive subject in the chaotic flow of the dream narrative. In contrast, the rare state of lucid dreaming is characterized by full-blown consciousness including all higher-order aspects: the dreamer regains metacognitive abilities and memory, becomes fully aware of his current state of consciousness, and experiences volition and fully realized agency (Metzinger, 2003; Windt and Metzinger, 2007). As phrased by Van Eeden (1913), who coined the term lucid dreaming: “the sleeper remembers his day-life and his own condition, reaches a state of perfect awareness, and is able to direct his attention, and to attempt different acts of free volition.”

On a closer look, however, the situation is less clear: on the one hand, during alert wakefulness, the experience of volitional capabilities may be strikingly impaired as seen, e.g., in pathological cases such as delusions of alien control in schizophrenic patients (Lafargue and Franck, 2009) or in alien hand syndrome (Biran and Chatterjee, 2004). On the other hand, during altered states of consciousness such as hypnosis (Oakley and Halligan, 2013) or even non-lucid REM sleep (Takahara et al., 2006), volitional behavior can be observed. Moreover, lucid dreaming is not an all-or-nothing phenomenon, but might occur in different degrees from pre-lucid reflections to full-blown lucid control dreams (Tyson et al., 1984; Barrett, 1992; Kahan and LaBerge, 1994). Descriptions of higher-order aspects of consciousness in lucid dreaming, including volitional capacities, rely mainly on anecdotal evidence, but have rarely been studied systematically. Two recent exceptions are the Metacognitive, Affective, Cognitive Experience questionnaire (MACE; Kahan and Sullivan, 2012) and the Lucidity and Consciousness in Dreams scale (LuCiD; Voss et al., 2013) which have been used to assess metacognition during different states of consciousness including lucid dreaming.

Here, we assessed different aspects of volition during normal dreaming, lucid dreaming, and wakefulness with an adapted version of the Volitional Components Questionnaire (VCQ; Kuhl and Fuhrmann, 1998) in lucid dreamers. We hypothesized that experienced volition would be generally higher in both wakefulness and lucid dreaming compared to non-lucid dreaming. We exploratively tested if the subscales of the VCQ would differentially vary between the three states of consciousness.

## MATERIALS AND METHODS

Ten healthy subjects (mean age 28.1 ± 9.8 years, age range 19–47 years, five female) recruited at the University of Munich or from a volunteer database of the Max Planck Institute of Psychiatry participated in this study. They all were experienced lucid dreamers with a reported mean frequency of 1.9 ± 0.7 lucid dreams per week. Lucid dreaming ability was verified in five of the subjects in a sleep laboratory with full polysomnographic recordings, exploiting the classical eye signaling technique (LaBerge et al., 1981). For the other five subjects, lucid dreaming ability was assessed by self-report.

To measure volition in different states of consciousness, we adapted the German short version of the VCQ (Selbststeuerungs-Inventar, SSI-K3; Kuhl and Alsleben, 2012). The VCQ is an instrument to measure different aspects of volitional competence; it specifically aims at assessing the subjective experience of volitional components supporting central coordination of goal-maintenance and self-maintenance (Kuhl and Fuhrmann, 1998). The short form includes 13 subscales consisting of four items each. The test subjects have to rate the extent to which the item applies to themselves on a four-point Likert scale from 1 (not at all) to 4 (wholly). The validity of both the long and short versions has been repeatedly demonstrated (Forstmeier and Rüddel, 2008). Since several of the 13 subscales are not meaningfully applicable to the dreaming state, as they, e.g., ask to evaluate time frames of several weeks, we chose to restrict the study to six subscales: *self-determination*, *planning ability*, *intention enactment*, *powers of concentration*, *self-access*, and *integration*. As a measure for the general experience of volitional capacity, an overall score consisting of the mean of all six subscales was calculated. Where necessary, questions were adapted to be applicable to the dreaming state, e.g., the integration subfactor item “On many days I feel the opposite of what I felt before” was changed to “I often feel the opposite of what I felt before.” The sequential order of questions was adopted from the original questionnaire.

Subjects were asked to complete the questionnaire at least once for each of the three states of consciousness: in the morning after awakening from a non-lucid dream, in the morning after awakening from a lucid dream, and after a normal day of wakefulness, i.e., before going to bed in the evening. Specifically, subjects were instructed to rate their general experience during the respective state, using the preceding dream or day as an anchor or reminder thereof. For those individuals who completed the questionnaire more than once for one of the three states of consciousness, we used the mean score of the given state for further analysis. For wakefulness, four subjects contributed multiple questionnaires adding up to a total of 15; for non-lucid dreaming, five subjects contributed multiple questionnaires adding up to a total of 20; for lucid dreaming, four subjects contributed multiple questionnaires adding up to a total of 14.

For statistical analysis of the VCQ overall score, we performed a repeated measures ANOVA with the three factor levels *non-lucid dreaming*, *lucid dreaming*, and *wakefulness*. For specific comparisons between the three states of consciousness, we performed post hoc two-sided paired *t*-tests. For statistical analysis of the six subscales, we first performed a repeated measures MANOVA with the three factor levels *non-lucid dreaming*, *lucid dreaming*, and *wakefulness*. For further analysis of the subscales that revealed significant results in the following ANOVAs, we subsequently performed two-sided paired *t*-tests to analyze specific differences between the states of consciousness. All significance levels were set at *p* = 0.05.

## RESULTS

The ANOVA for the VCQ overall score revealed a significant effect of the state of consciousness (*F*_2,18_ = 4.4, *p* = 0.027, *η*^2^ = 0.33). Subsequent *t*-tests demonstrated that both wakefulness (*t*_9_ = 2.7, *p* = 0.026, *r* = 0.66) and lucid dreaming (*t*_9_ = 2.3, *p* = 0.044, *r* = 0.61) differed from non-lucid dreaming, however not from each other (*t* = 0.5, *p* = 0.618, *r* = 0.17). Hence, volition was strongly experienced during wakefulness and lucid dreaming, but considerably less so during non-lucid dreaming. For comparisons of the overall results, see **Table 1**.

**Table 1:** Experienced volition during wakefulness, lucid dreaming and non-lucid dreaming according to the Volitional Components Questionnaire (VCQ) overall and subscale results.

Scale	Wakefulness	Lucid dreaming	Non-lucid dreaming
Overall	**2.93 ±0.11**	**2.85 ±0.14**	2.47 ±0.13
Self-determination	**3.10 ±0.17**	**3.33 ±0.14**	2.21 ±0.16
Planning ability	**2.81 ±0.19**	1.40 ±0.14	1.29 ±0.13
Intention enactment	2.43 ±0.20	**3.43 ±0.15**	2.49 ±0.25
Powers of concentration	2.65 ±0.22	2.67 ±0.34	2.76 ±0.33
Self-access	3.35 ±0.18	3.23 ±0.28	3.03 ±0.25
Integration	3.24 ±0.18	3.06 ±0.26	3.06 ±0.21

*Bold print indicates significantly higher volition compared to non-bold printed data. Data are given as mean scores ±SEM, for statistics see results section.*

The MANOVA for the subscales of the VCQ revealed a significant effect of the state of consciousness (*F*_12,2__6_ = 8.9, *p* < 0.001, *η*^2^= 0.80), which turned out to be significant for the subscales *self-determination* (*F*_2,18_ = 15.4, *p* < 0.001, *η*^2^ = 0.63), *planning ability* (*F*_2,18_ = 31.2, *p* < 0.001, *η*^2^ = 0.78), and *intention enactment* (*F*_2,18_ = 7.3, *p* = 0.007, *η*^2^= 0.45), but not for *powers of concentration* (*F*_2,18_ = 0.1, *p* = 0.942, *η*^2^= 0.01), *self-access* (*F*_2,18_ = 0.6, *p* = 0.573, *η*^2^ = 0.06), or *integration* (*F*_2,18_ = 0.7, *p* = 0.503, *η*^2^ = 0.07). Subsequent *t*-tests demonstrated that *self-determination* was significantly more pronounced during both wakefulness (*t*_9_ = 5.6, *p* < 0.001, *r* = 0.88) and lucid dreaming (*t*_9_ = 5.2, *p* < 0.001, *r* = 0.87) compared to non-lucid dreaming, however did not differ between the two former states of consciousness (*t*_9_ = 0.9, *p* = 0.461, *r* = 0.28). *Planning ability* was most pronounced during wakefulness compared to both lucid (*t*_9_ = 6.5, *p* < 0.001, *r* = 0.91) and non-lucid dreaming (*t*_9_ = 5.7, *p* < 0.001, *r* = 0.88), but did not differ between the latter two states (*t*_9_ = 0.8, *p* = 0.407, *r* = 0.25). *Intention enactment* turned out to be most pronounced during lucid dreaming compared to both wakefulness (*t*_9_ = 3.9, *p* = 0.004, *r* = 0.79) and non-lucid dreaming (*t*_9_ = 3.2, *p* = 0.011, *r* = 0.73), while the latter two states did not differ from each other (*t*_9_ = 0.2, *p* = 0.862, *r* = 0.06). For subscale comparisons, see **Table 1**.

## DISCUSSION

Comparing the experience of volition as assessed by the VCQ during three different states of consciousness, we found volition to be generally most pronounced during both wakefulness and lucid dreaming as compared to non-lucid dreaming. A more differential picture appeared when the subscales of the VCQ were analyzed separately.

For both lucid dreaming and wakefulness, *self-determination* was rated higher than for non-lucid dreaming. This subscale is probably the most prototypical volitional component, asking to what degree the subject experiences being able to act freely according to his will. The fact that the result of this subscale is in line with the overall score confirms the hypothesis that volition is generally more pronounced during both wakefulness and lucid dreaming compared to non-lucid dreaming.

For wakefulness, *planning ability* was rated higher than for both lucid and non-lucid dreaming. This subscale asks for how well organized the subject pursues his plans and intentions. The fact that this subfactor is most pronounced during wakefulness compared to both dreaming states might be interpreted as a sign for a more spontaneous execution of intentions during dreaming.

For lucid dreaming, *intention enactment* was rated higher than for both wakefulness and non-lucid dreaming. This factor asks for how promptly and determined intentions are executed. On first sight, this seems to be a surprising finding, demonstrating that a component of volition is more strongly experienced during a state of sleep than during wakefulness. However, on second sight a strong feeling of being able to enact one’s intentions during lucid dreaming seems reasonable, as the dreamer is aware that in contrast to the constraints of waking life, during dreams all potential obstacles are not real and hence can easily be overcome. This interpretation would also be in line with the former finding of a comparably low level of experienced planning ability during lucid dreaming: organized planning might be possible during lucid dreaming in principle, however is rarely actually performed since intention execution is possible without such effort.

Neither *powers of concentration*, nor *self-access*, nor *integration* differed between the three states of consciousness. The first of these subfactors asks for how easily the subject gets distracted from his current line of intentional thought. The failure to find any difference between the three states of consciousness is rather surprising, since concentration and goal-directed thinking are generally thought to be strongly impaired during non-lucid dreaming (Hobson and Pace-Schott, 2002; Metzinger, 2003). The subfactor *self-access* asks for the quality of access to one’s intentions and feelings in stressful situations. It might be speculated that in such situations, also during wakefulness and lucid-dreaming, self-reflection might be impaired, thereby leveling potential differences of self-access that would occur in non-stressed situations. The subfactor *integration* asks for the occurrence of seemingly contradictory behaviors and emotions. It is rather surprising that non-lucid dreaming does not differ from the other two states, since incongruities and inconsistencies are generally associated most strongly with the dreaming state (Mamelak and Hobson, 1989). However, such inconsistencies are typically attributed to the dream plot rather than to the dreamer, whose mental complexity is narrow and “single-minded” compared to a much broader repertoire of behaviors and thoughts experienced during wakefulness or lucid dreaming (Rechtschaffen, 1978). Hence, compared to a bizarre and highly incongruent dream plot, the single-mindedness of dream cognition might be experienced as relatively straightforward.

In the following, we will try to embed the topic of volition in a broader discussion of the multiple facets and neural correlates of human consciousness.

### BASAL VS. HIGHER-ORDER ASPECTS OF CONSCIOUSNESS

The enquiry into consciousness has long been the domain of philosophy, however recent years witnessed a growing interest also among neuroscientists in the problems surrounding consciousness. While there is still little agreement on a specific characterization or definition, it seems clear that consciousness is a multifaceted concept, with its different aspects varying dramatically between species, vigilance states, or health conditions. A common categorization differentiates between basal and higher-order aspects of consciousness: the concept of basal (or primary) consciousness comprises perceptions and emotions, whereas higher-order (or secondary) consciousness is proposed to constitute reflections on these (for a review cf. Morin, 2006). As phrased by Edelman (2003, p. 5521):“Higher-order consciousness allows its possessors to go beyond the limits of the remembered present of primary consciousness. An individual’s past history, future plans, and consciousness of being conscious all become accessible.”

A striking variation in consciousness is experienced every day during the sleep–wake cycle: awake human subjects are normally alert, aware of external and internal stimuli, and able to reflect on their perceptions and emotions and to volitionally act according to their intentions. These experiences and capabilities fade during the process of falling asleep, however the progress through the sleep cycle is associated with a reinstatement of essential features of consciousness: REM sleep evokes the most vivid and intense dreams, in which the sleeper perceives a hallucinated environment and often experiences strong emotions.

However, the dreaming state instantiates only basal aspects of consciousness, being deficient in reflective thought, metacognition and volitional capabilities: the internally generated perceptions and emotions experienced during dreaming typically show many cognitive abnormalities, with a bizarre dream plot full of gaps, delusional thought, and a complete lack of insight into the current condition (Hobson and Pace-Schott, 2002; Metzinger, 2003). Rechtschaffen (1978) called this persistence of a single train of related thoughts and images without disruption from other simultaneous thoughts or reflections the “single-mindedness” of dreams. He pointed out that without reflectiveness, there could hardly be volitional control. Nevertheless, some rudimentary processes of reflection and volition have been reported to occur during dreaming (Kahan et al., 1997; Wolman and Kozmová, 2007), even though less often than for waking episodes (Kahan et al., 1997; Voss et al., 2013). Our results confirm these findings, suggesting a generally weaker experience of volition during non-lucid dreaming compared to wakefulness, however with some components being similarly expressed during wakefulness and dreaming.

### LUCID DREAMING AS HIGHER-ORDER CONSCIOUSNESS

In contrast to the restricted consciousness of normal dreaming, the rare state of lucid dreaming is characterized by full-blown consciousness including all higher-order aspects: the sleeping subject is no longer deluded by the dream narrative, but becomes fully aware of the true nature of his current state of consciousness (LaBerge et al., 1981). This wake-like intellectual clarity comprises a restored access to memory functions including increased availability of self-related information, and fully realized agency, enabling the dreamer to volitionally execute his intentions within the dream narrative (Metzinger, 2003; Windt and Metzinger, 2007). Lucid dreaming can be trained (LaBerge, 1980; Purcell et al., 1986), which makes this phenomenon a promising research topic despite its rarity in untrained subjects (Schredl and Erlacher, 2011).

In comparing lucid and non-lucid REM sleep, the distinction between basal and higher-order consciousness is of great value, since the contrast between lucid and non-lucid dreaming strikingly mirrors the conceptual contrast between basal and higher-order consciousness (Dresler et al., 2009; Hobson, 2009): while all basal features of consciousness like perceptions and emotions are present in normal dreaming, metacognitive reflections and the insight into the current state of consciousness is – by definition – bound to lucidity. Since also in non-lucid dream reports some reflective thoughts have been reported and since also during daydreaming and other phases of wakefulness active reflections are frequently absent, it has been argued that metacognitive activity differs only quantitatively and not qualitatively between dreaming and waking consciousness (Kahan et al., 1997; Kahan and LaBerge, 2011). However, this absence is only a “local,” not global feature of such phases: it is hardly imaginable, at least for non-pathological cases, that the day-dreaming subject misinterprets the daydream for reality once paying attention to his current state. For the dreaming state, in contrast, this is completely normal – unless the dreamer eventually achieves lucidity through these “prelucid” reflections (Tyson et al., 1984).

Lucid dreaming may even be critical to fully understanding the neural correlates of higher-order consciousness, because in contrast to, e.g., coma–wake, anesthesia–wake, or sleep–wake comparisons, there is no major shift in vigilance state as defined by formal neurophysiological criteria: lucid REM sleep still is REM sleep proper according to the classical Rechtschaffen and Kales (1968) or new AASM (Iber et al., 2007) sleep scoring criteria. When compared to wakefulness, pathological or pharmaceutically induced loss of consciousness also reduces the brain’s basal metabolism, as does deep sleep. Dreaming therefore provides the only phenomenon we know of, that can contrast basal consciousness with full-blown higher-order consciousness within the same vigilance level (Spoormaker et al., 2010), allowing for comparison of cerebral activity by means of EEG, PET, or fMRI without differences in the basal activity state.

### NEURAL CORRELATES OF LUCID DREAMING

On the phenomenological level, REM sleep is the sleep stage associated with the most vivid sleep mentation (Fosse et al., 2001). On the neurobiological level, it is associated with strong activation of visual association areas and limbic structures such as the amygdala, while the dorsolateral prefrontal cortex (DLPFC) and parietal areas are deactivated (Maquet et al., 1996; Braun et al., 1998). This activation pattern has been proposed to underlie the visual hallucinations, emotional intensifications, and metacognitive impairments experienced in most dreams (Hobson and Pace-Schott, 2002; Schwartz and Maquet, 2002). In particular diminished activity in the DLPFC during REM sleep has been related to cognitive aspects of dreaming such as impaired directed thought, volitional control, and a complete lack of insight into the current state of consciousness (Hobson and Pace-Schott, 2002; Schwartz and Maquet, 2002).

In contrast to normal dreaming, the regaining of wake-like metacognitive capabilities during lucid dreaming is related to increased EEG gamma-band activity over dorsolateral prefrontal areas (Voss et al., 2009). fMRI data have confirmed increased activation of the DLPFC during lucid dreaming, as well as of bilateral frontopolar and parietal areas (Dresler et al., 2012). These brain regions have been related to self-focused metacognitive evaluation (Stuss et al., 2001; Schmitz et al., 2004), supervisory modes (Burgess et al., 2007), and self-referential processing in general including the experience of agency (Cavanna and Trimble, 2006). Their activation during lucid dreaming is in line with the notion that lucidity consists in an increased availability of self-related information, leading to a much higher degree of coherence and stability of the phenomenal self during lucid dreaming (Metzinger, 2003). Our findings fit well into this literature, demonstrating that volition is similarly experienced during wakefulness and lucid dreaming as compared to non-lucid dreaming.

### NEURAL CORRELATES OF VOLITION

As is the case for consciousness, volition is a multifaceted concept, hence not traceable to one specific brain region. However, several cortical areas have repeatedly been demonstrated to be related to volitional processes. While most studies show motor areas to be involved in volitional action, this research mainly focuses on willed motor actions (Haggard, 2008), which seem to share similar neural substrates during wakefulness and dreaming (Erlacher and Schredl, 2008; Dresler et al., 2011). In contrast, more general or abstract intentions are thought to rely on the dorsolateral prefrontal cortex (Roskies, 2010). In addition, early stages of intentional action have been related to anterior prefrontal brain regions. Such processing of complex information, only broadly determined by specific task demands, is then thought to travel posteriorly to enter later stages of intentional action (Brass et al., 2013). The subjective experience of volitional agency has been associated with parietal brain regions (Roskies, 2010). Hence, in line with our findings, general aspects of volitional control and the subjective experience thereof rely on brain regions that are highly active during lucid compared to non-lucid dreaming.

### CONSCIOUSNESS IN HUMANS AND NON-HUMAN ANIMALS

**FIGURE 1:**
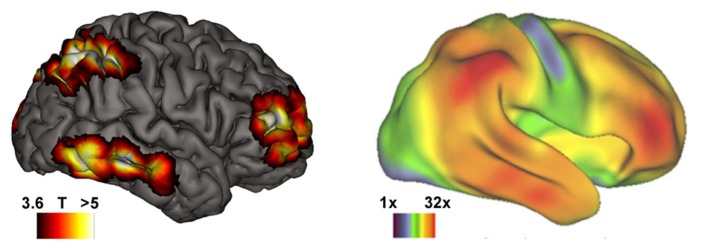
**Brain areas subserving the transition from basal to higher-order consciousness in REM sleep dreaming mirror those with strongest volumetric expansion in humans compared to non-human primates**. Left: during lucid dreaming, the dorsolateral prefrontal and frontopolar cortices, parietal lobules, and inferior/middle temporal gyri among other cortical regions are strongly activated as compared with non-lucid REM sleep (republished with permission of the American Academy of Sleep Medicine, from Dresler et al. (2012); permission conveyed through Copyright Clearance Center, Inc.). Right: neuroanatomical differences between humans and non-human primates (Van Essen and Dierker, 2007; reprint with permission of Cell Press). Color-coded are regional volumetric expansions in the human relative to the macaque brain hot colors depict up to a 32-fold volumetric increase in humans. Right lateral view.

Higher-order aspects of consciousness are traditionally thought to be most pronounced in humans (Edelman, 2001). In particular volitional capabilities have been proposed to be a distinctive human attribute (Dijksterhuis and Aarts, 2010; Frith, 2013). If the contrast between ordinary and lucid dreaming mirrors that between basal and higher-order consciousness, data on the neural correlates of dream lucidity might shed new light on this debate. Indeed it turns out that cerebral regions showing increased activity during lucid dreaming also show extensive volumetric expansion in humans as compared to non-human primates (Van Essen and Dierker, 2007; see **Figure 1**). Recently the hypothesis was proposed that only animals possessing higher-order aspects of consciousness may develop psychotic states – “in other words an animal needs to have a highly developed mind in order to go out of it” (Hobson and Voss, 2011, p. 993). Neuroimaging data on lucid dreaming support this claim: areas activated during lucid dreaming (Dresler et al., 2012) do not only mirror human vs. non-human primate brain differences (Van Essen and Dierker, 2007), but also show striking overlap with brain areas associated with insight deficits in psychosis (Dresler et al., in revision).

### LIMITATIONS

A couple of limitations have to be kept in mind for the interpretation of our study’s results. First, we used an adapted version of VCQ that was not specifically validated for its use in different states of consciousness. This applies in particular to the overall score combining the six subscales. While the original version was created to evaluate time frames of several weeks, for our adapted version only items were chosen that are applicable to shorter episodes like dreams. Second, whereas the ratings for lucid and non-lucid dreaming were collected after awakening from the respective dream phase in the early morning, the ratings for wakefulness were collected after a full day of wakefulness in the evening. Thus, the length of the rated episodes differed, and it cannot be excluded that chronobiological influences affected the ratings. Third, the order of data collection was not randomized, but started for all subjects with the wakefulness ratings, followed by the non-lucid dreaming ratings, which were finally followed by the lucid dreaming ratings. Thus, order effects might have influenced the results. However, several subjects completed more than one questionnaire and did so after a complete round of ratings. Since the scores from these repeated ratings did not differ from the first ratings, it is rather unlikely that rating order affected the results. Fourth, gender differences for content and recall have been reported for non-lucid (Schredl et al., 2004) and lucid (Schredl and Erlacher, 2011) dreams, however our small sample size does not allow a reliable analysis of possible gender effects on state-dependent volition. An explorative analysis did not reveal gender effects (*p* > 0.2) or gender × state interactions (*p* > 0.4).

### CONCLUSION AND FUTURE DIRECTIONS

Our study confirmed the multifaceted nature of consciousness: volitional components of higher-order consciousness are differentially expressed among different conscious states. On a coarser level, the generally wake-like expression of volition during lucid dreaming is well in line with the neural activity pattern observed during this state. Up until 15 years ago, using lucid dreaming for the study of consciousness was not seen as experimentally advantageous (Crick and Koch, 1998). However, neuroimaging research into the neural correlates of lucid dreaming and its association with metacognitive and volitional processes has proven lucid dreaming to be a highly promising approach for the investigation of higher-order aspects of consciousness. Neural correlates of lucid dreaming show a remarkable overlap with areas and networks subserving self-reflective thought and volitional capabilities. In addition, these areas show the strongest differences between human and non-human primates, strengthening suggestions that higher-order aspects of consciousness are most pronounced in humans.

While research into lucid dreaming is currently hampered by the rarity of the phenomenon, systematic training (Stumbrys et al., 2012), and new technical approaches for its induction like transcranial direct current stimulation (tDCS; Noreika et al., 2010; Stumbrys et al., 2013) might lead to research programs beyond a collection of case studies. In such research programs, subjects might be asked to actively engage in metacognitive processes and volitional acts during lucid dreaming, thereby tracing higher-order consciousness from its state-dependent absence to the regaining of the ability to engage in higher-order conscious thought to its actual execution. Using neuroimaging methods in combination with refined measures of the degree of lucidity, e.g., by exploiting scales that assess several dimensions of volition and insight during dreams (Voss et al., 2013), the specific involvement of several brain regions in distinct higher-order aspects of consciousness may be disentangled. Such studies would further refine the neural correlates underlying the multiple facets of human consciousness.

## Conflict of Interest Statement

The authors declare that the research was conducted in the absence of any commercial or financial relationships that could be construed as a potential conflict of interest.
